# Prediction of early vascular cement leakage following percutaneous vertebroplasty in spine metastases: the Peking University First Hospital Score (PUFHS)

**DOI:** 10.1186/s12885-021-08503-2

**Published:** 2021-07-02

**Authors:** Xuedong Shi, Yunpeng Cui, Yuanxing Pan, Bing Wang, Mingxing Lei

**Affiliations:** 1 Department of Orthopedic Surgery, Peking University First Hospital, No.8 Xishiku Street, Xicheng District, Beijing, 100032 China; 2 Department of Orthopedic Surgery, Hainan Hospital of Chinese PLA General Hospital, Haitang District, Jianglin Rd, Sanya, 572013 China; 3 Graduate School of Chinese PLA Medical College, No. 28 Fuxing Road, Haidian District, Beijing, 100853 China

**Keywords:** Spine metastases, Percutaneous vertebroplasty, Vascular cement leakage, Prediction score

## Abstract

**Background:**

Cement leakage into venous blood posed significant challenge to surgeons. The aim of the study was to create a Peking University First Hospital Score (PUFHS) which could evaluate the probability of vascular cement leakage among spine metastases patients following percutaneous vertebroplasty.

**Methods:**

The study retrospectively enrolled 272 spine metastases patients treated with percutaneous vertebroplasty. We randomly extracted all enrolled patients as the training or validation group and baseline characteristic comparison was assessed between the two groups. Creation of the PUFHS was performed in the training group and validation of the PUFHS was performed in the validation group.

**Results:**

Of all the 272 patients, the total number of included vertebrae was 632 and the median treated levels were 2 per patient. Vascular cement leakage occurred in 26.47% (72/272) of patients. The baseline characteristics were comparable between the two groups (*P* > 0.05). Three risk predictors (primary cancer types, number of treated vertebrae levels, and vertebrae collapse) were included in the PUFHS. The area under the receiver operating characteristic curve (AUROC) of the PUFHS was 0.71 in the training group and 0.69 in the validation group. The corresponding correct classification rates were 73.0 and 70.1%, respectively. The calibration slope was 0.78 (95% confidence interval[CI]: 0.45–1.10) in the training group and 1.10 (95% CI: 0.73–1.46) in the validation group. The corresponding intercepts were 0.06 (95% CI: − 0.04–0.17) and − 0.0079 (95% CI: − 0.11–0.092), respectively.

**Conclusions:**

Vascular cement leakage is common among spine metastases after percutaneous vertebroplasty. The PUFHS can calculate the probability of vascular cement leakage, which can be a useful tool to inform surgeons about vascular cement leakage risk in advance.

**Supplementary Information:**

The online version contains supplementary material available at 10.1186/s12885-021-08503-2.

## Background

Spine metastases occur in 5–10% of all malignant tumors during disease course [1], and the incidence of spine metastases is also increasing as systemic therapies for cancer patients improve. Spine metastases are often characterized by severe back pain and vertebral compression fractures, which may lead to declining mobility, kyphosis, and even neurologic compression [2]. If those patients are left untreated, they may suffer from poor quality of life.

The therapeutic strategies for spine metastases are usually conventional palliative, aiming at improving patient’s quality of remaining life and emphasizing in reducing pain and improving or, perhaps at most cases, just maintaining function status. Although open surgery is capable of realizing fully decompression and/or total removal of spine metastatic lesions, it can also bring out large trauma, severe complications, and delays in systematic treatment of the primary tumor. Notably, it is not appropriate for multiple spine metastases to receive open surgery [3]. Therefore, percutaneous vertebroplasty, a minimally invasive procedure, was developed to treat spine metastases, which has become one of the fastest emerging procedures in spine surgery [4]. This technique consists of percutaneous injection of polymethylmethacrylate (PMMA) into vertebrae body through transpedicular approach, which is proved to be an effective technique for promptly obtaining pain relief, controlling local tumor burden, preventing further vertebral collapse or spinal cord compression, and facilitating the return to early systemic and radiation therapy [5, 6].

However, despite the fact that percutaneous vertebroplasty is minimal invasive, complications can still occur during surgery. PMMA leakage is considered as the major cause of complications. Cement leakage into venous blood vessels and/or the spinal canal may result in serious consequences, though the majority of cement leakage would not cause any symptoms [7]. Pulmonary embolism can be caused by PMMA leaking into blood vessels and the incidence varies from 4.6 to 23.0% [8]. A systematic review revealed serious complication rates ranged from 2 to 11.5% [9]. Intracardiac cement embolism could be up to 3.9% [10]. Literature reported leaked cement pulmonary embolism and cardiac perforation was regarded as a cause of unexpected death following percutaneous vertebroplasty [11]. Thus, appropriate strategies to guide PMMA injections are really warranted to prevent vascular cement leakage and subsequent life-threatening complications.

Therefore, the aim of the study was to identify potential risk factors for predicting vascular cement leakage in spine metastases following percutaneous vertebroplasty and further create the Peking University First Hospital Score (PUFHS) to evaluate the probability of vascular cement leakage so as to realize early detection of this complication.

## Methods

### Patients

We retrospectively enrolled 272 spine metastases patients treated with percutaneous vertebroplasty at the orthopedic department of the Peking University First Hospital between January 2010 and January 2019. Patients were included if they (1) had an age of more than 50 years old, (2) had mixed or osteolytic metastatic vertebrae lesions, (3) had serious or uncontrolled back pain, and (4) received percutaneous vertebroplasty; Patients were excluded if they (1) had intramedullary metastases, (2) vertebrae compression fracture due to primary spine tumor, trauma, osteoporosis, and/or angioma, (3) received combined therapy (i.e thermal ablation + vertebroplasty), and (4) skin infections at patient’s corresponding involved vertebrae. If a patient’s serious uncontrolled back pain was not significantly relieved after conservative treatments, they were also considered to be performed with percutaneous vertebroplasty; If patients had severe radiculopathy and deteriorated function and were tolerable to open surgery, decompressive surgery and spine stabilization was recommended to these patients; If patients performed with more than one percutaneous vertebroplasty at different time, the second and subsequent operations were not analyzed in the study. This study was performed based on the Declaration of Helsinki. The Ethics Committee Board of the Peking University First Hospital approved the study and waived patient’s consent form due to anonymized and retrospective data.

### Study design

We randomly extracted 3/4 of all included patients as the training group (*n* = 204) or validation group (*n* = 204), respectively. Baseline characteristic comparison was evaluated between the training and validation group. Creation of the PUFHS was performed in the training group and the validation of the PUFHS was performed in the validation group.

### Primary outcome

**Fig. 1:**
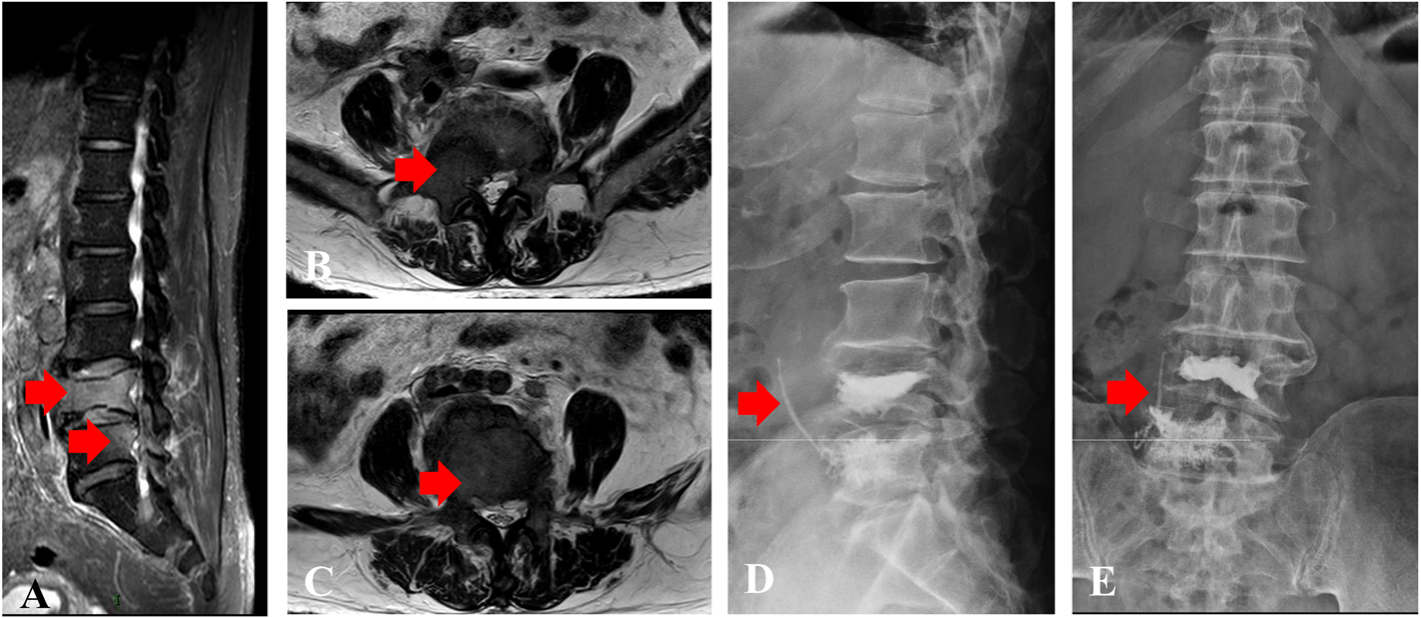
An 80-year-old female with spine metastases treated with percutaneous vertebroplasty. **A** Preoperative sagittal lumbar vertebra MRI (T2) showed spine metastases at L4 and L5; **B** Preoperative transversal MRI (T2) showed metastatic lesion at L4; C. Preoperative transversal MRI (T2) showed metastatic lesion at L5; The red arrow indicates metastatic tumor in (**A**, **B**, and **C**). **D** Postoperative lateral lumbar vertebra X-ray presented bone cement at L4 and L5 and vascular cement leakage; **E** Postoperative anteroposterior lumbar vertebra X-ray presented bone cement at L4 and L5 and vascular cement leakage. The red arrow indicates vascular cement leakage in (**D** and **E**)

Vascular cement leakage was defined that cement leaks into veins, including anterior external venous plexus or basivertebral veins, which was evaluated using intraoperative fluoroscopy images (mobile C-arm) or postoperative CT examination and X-ray. Cement pulmonary embolism was a particular type of the vascular cement leakage which was assessed based on chest radiographs and/or CT examinations, if patient’s pulmonary symptoms were presented after surgery. If patients had radicular pain, neurological deficits, and/or dyspnea because of vascular cement leakage, researchers would record the corresponding clinical symptoms. Figure 1 shows a case report. The bone cement mainly used in the study was Mendec Spine Resin and Kit (Tecres S.P.A. Italy).

### Potential risk predicators

Eleven potential risk predictors were collected and analyzed for evaluating vascular cement leakage in spine metastases following percutaneous vertebroplasty. The potential risk factors included basic information, such as age (mean, years), gender (male vs. female), primary cancer types (rapid vs. moderate vs. slow) [12], therapeutic information, including preoperative treatments (topical treatments vs. systematic treatments vs. no treatment), and radiographic data, such as the number of treated vertebrae levels (1 vs. 2 vs. 3 vs. ≥4), vertebrae collapse (no collapse vs. less than 50% vs. more than 50%) [13], cortical osteolytic destruction in posterior wall (yes vs. no), vertebral endplate fracture (yes vs. no), the Bilsky scale (0 vs. 1 vs. 2 vs. 3) [14], appearance of spine metastases (mixed lesions vs. osteolytic lesions), and load-bearing lines of spine (normal vs. abnormal). Patient’s age was defined as the time interval between patient’s birth data and surgery data. Rapid growth cancers were defined as patients diagnosed with lung cancer, esophageal cancer, stomach cancer, liver cancer, pancreatic cancer, colon cancer, and unknown cancer, moderate growth cancers were kidney cancer and uterus caner, and slow growth cancers were breast cancer, thyroid cancer, prostate cancer, and others. The Bilsky scale was used to evaluate the severity of spinal cord compression: a higher score represented a severer spinal cord compression. Preoperative treatments were classified into topical treatments (topical analgesics and radiotherapy), systematic treatments (oral or intravenous analgesics, targeted drugs, and chemotherapy), and no treatment.

### Creating the PUFHS

In the training group, the Least Absolute Shrinkage and Selection Operator (LASSO) method was used to identify the above-mentioned potential risk predictors, and significant predictors were included in the PUFHS. The coefficients of the included risk predictors were assigned based on the estimates obtained from the multiple logistic regression analysis. The PUFHS would be created as follows: P (Y = 1) = *e*^*intercept* + *ax*1 + *bx*2 + *c*x3^ / (1+ *e*^*intercept* + *ax*1 + *bx*2 + *c*x3^). In the PUFHS, a, b, and c were coefficients of the included risk predictors. Intercept was also calculated according to the multiple logistic regression analysis. P (Y = 1) represented the predicted probability of vascular cement leakage.

### Validating the PUFHS

Validation of the PUFHS was performed in the validation and training group. The discrimination and calibration ability was used to evaluate the predictive performance of the PUFHS. The discrimination ability was defined as the capability that the PUFHS could distinguish patients with vascular cement leakage from patients without this complication. The calibration ability was defined as the consistence between the PUFHS-predicted probability of vascular cement leakage and the actual observed probability of vascular cement leakage.

The PUFHS’s discrimination ability was evaluated using the area under the receiver operating characteristic (AUROC) curve and discrimination slope. A C-value of more than 0.6 from the AUROC curve indicated useful model and 0.7 indicated good model. Discrimination slope was the difference between the PUFH-predicted mean probability with vascular cement leakage (positive events) and without it (negative events).

The calibration ability of the PUFHS was evaluated using the calibration slope and goodness-of-fit test. Calibration slope was measure by plotting deciles of the predicted probability of vascular cement leakage against the observed proportions in each decile. A smooth line (Y = ax + b) was fitted using linear regression analysis in the Microsoft Excel software. In the smooth line, ‘a’ indicated the calibration slope and ‘b’ indicated the intercept. In ideal circumstance, the closer calibration slope is to 1, the better it is; the closer intercept is close to 0, the better it is. A *P*-value of more than 0.05 obtained from the goodness-of-fit text indicated good calibration.

### Statistical analysis

Analyses were performed in SAS 9.2 software and R version 3.5.3 for Windows XP. Continuous variables were presented as mean ± SD. The characteristic differences between the training and validation group were analyzed based on the chi-square test and *t* or rank test. The difference between the three risk groups was compared using the Kruskal Wallis test and the Chi-square test. The calibration slope and intercept was calculated using the Microsoft Excel software. A *P*-value of less than 0.05 was considered as statistical significance.

## Results

### Patient’s demographics

Of all the 272 patients, the mean age was 67.92 ± 10.21 years (Table 1). The majority of patients were male (59.93%, 163/272) and diagnosed with rapid growth cancers (56.25%, 153/272), followed by slow growth cancers (28.31%, 77/272). In detail, 33.82% (92/272) of patients had lung cancer, 16.18% (44/272) had prostate cancer, and 11.40% (31/272) had renal cancer. The number of patients received topical treatments (36.40%, 99/272) was similar with patients treated with systematic treatments (32.72%, 89/272) or no treatment (30.88%, 84/272).

**Table 1:** Patient’s basic characteristics

Characteristics	Patients (*n* = 272)
Age (years, mean ± SD)	67.92 ± 10.21
Gender	
Male	59.93% (163/272)
Female	40.07% (109/272)
Primary cancer types	
Slow growth	28.31% (77/272)
Moderate growth	15.44% (42/272)
Rapid growth	56.25% (153/272)
Preoperative treatments	
Topical treatments	36.40% (99/272)
Systematic treatments	32.72% (89/272)
No treatment	30.88% (84/272)
Number of treated vertebrae levels	
1	37.87% (103/272)
2	26.10% (71/272)
3	16.18% (44/272)
≥ 4	19.85% (54/272)
Vertebrae collapse	
No collapse	61.76% (168/272)
Less than 50%	24.63% (67/272)
More than 50%	13.60% (37/272)
Cortical osteolytic destruction in posterior wall	
Yes	41.91% (114/272)
No	58.09% (158/272)
Vertebral endplate fracture	
Yes	17.65% (48/272)
No	82.35% (224/272)
The Bilsky scale	
0	77.21% (210/272)
1	10.29% (28/272)
2	10.66% (29/272)
3	1.84% (5/272)
Appearance of spine metastases	
Mixed lesions	17.65% (48/272)
Osteolytic lesions	82.35% (224/272)
Load-bearing lines of spine	
Normal	88.24% (240/272)
Abnormal	11.76% (32/272)
Vascular cement leakage	
Yes	26.47% (72/272)
No	73.53% (200/272)
The total number of included vertebrae	632
Sites of vertebra	
Cervical	1.27% (8/632)
Thoracic	46.04% (291/632)
Lumbar	46.04% (291/632)
Sacral	6.65% (42/632)

*Abbreviations*: *SD* standard deviation

Regarding radiographic data, the total number of included vertebrae was 632 and among them 1.27% (8/632) was cervical vertebra, 46.04% (291/632) was thoracic vertebra, 46.04% (291/632) was lumbar vertebra, and 6.65% (42/632) was sacral vertebrae. The majority of patients had only one treated vertebrae (37.87%, 103/272), no vertebrae collapse (61.76%, 168/272), complete vertebrae posterior wall (58.09%, 158/272), no vertebral endplate fracture (82.35%, 224/272), a Bilsky scale of 0 (77.21%, 210/272), osteolytic lesions (82.35%, 224/272), and normal load-bearing lines of spine (88.24%, 240/272). Vascular cement leakage occurred in 26.47% (72/272) of patients. Among them, only 1/72 patients with vascular cement leakage was symptomatic with the patient reporting mild dyspnea.

### Comparisons between the training and validation group

We randomly extracted 3/4 of all included patients as the training (*n* = 204) and validation group (*n* = 204), respectively. Table 2 shows baseline characteristic comparison between the two groups, which demonstrated that the distribution of the eleven potential risk predictors was similar and comparable. Regarding the primary outcome, 27.94% (57/204) of patients had vascular cement leakage in the training group and 25.00% (51/204) in the validation group (*P* = 0.50).

**Table 2:** The characteristic comparison of patients in the training and validation group

Characteristics	The training group (*n* = 204)	The validation group (*n* = 204)	*P*-values
Age (years, mean ± SD)	67.78 ± 10.12	67.85 ± 10.44	0.95
Gender			
Male	121	129	0.42
Female	83	75	
Primary cancer types			
Slow growth	56	60	0.90
Moderate growth	31	31	
Rapid growth	117	113	
Preoperative treatments			
Topical treatments	74	72	0.98
Systematic treatments	70	71	
No treatment	60	61	
Number of treated vertebrae levels			
1	76	80	0.77
2	47	53	
3	36	31	
≥ 4	45	40	
Vertebrae collapse			
No collapse	121	124	0.77
Less than 50%	56	50	
More than 50%	27	30	
Cortical osteolytic destruction in posterior wall			
Yes	86	88	0.84
No	118	116	
Vertebral endplate fracture			
Yes	39	37	0.80
No	165	167	
The Bilsky scale			
0	157	156	0.96
1	22	20	
2	20	23	
3	5	5	
Appearance of spine metastases			
Mixed lesions	33	37	0.60
Osteolytic lesions	171	167	
Load-bearing lines of spine			
Normal	180	176	0.55
Abnormal	24	28	
Vascular cement leakage			
Yes	57	51	0.50
No	147	153	
The total number of included vertebrae	495	467	N.A.
Sites of vertebra			
Cervical	7	5	0.93
Thoracic	241	222	
Lumbar	218	210	
Sacral	29	30	

*Abbreviations*: *SD* standard deviation, *N.A.* not applicable

### Creation of the PUFHS

In the training group, the LASSO method found that three of the eleven risk predictors, including primary cancer types, number of treated vertebrae levels, and vertebrae collapse, were significant and included in the PUFHS (Table 3). The coefficients of the three included predictors were assigned based on the estimates obtained from the multiple logistic regression analysis. Thus, the PUFHS was created as follows: P (Y = 1) = *e*^−2.67 + 0.26*x*1 + 0.65*x*2 − 0.30x3^ / (1+ *e*^−2.67 + 0.26*x*1 + 0.65*x*2 − 0.30x3^). ‘ 1’ indicates primary cancer types, ‘ *x* 2’ indicates number of treated vertebrae levels, and ‘ *x* 3’ indicates vertebrae collapse. The scores of the three predictors were assigned based on the original data. For example, if a spine metastasis patient with rapid growth cancer (3 points) had more than 50% vertebrae collapse (3 points) and one treated vertebra level (1 point), the vascular cement leakage probability of the patient was P (Y = 1) = *e*^−2.67 + 0.26 ∗ 3 + 0.65 ∗ 1 − 0.30 ∗ 3^ / (1+ *e*^−2.67 + 0.26 ∗ 3 + 0.65 ∗ 1 − 0.30 ∗ 3^) = 10.53%. We further developed a calculator which can calculate the probability of vascular cement leakage in order to facilitate the clinical utility of the score (Supplementary material).

**Table 3:** The PUFHS

Characteristics included in the PUFHS^a^	Scores	Estimates^b^
Intercept		− 2.67
Primary cancer types		
Slow growth	1	0.26
Moderate growth	2	
Rapid growth	3	
Number of treated vertebrae levels		
1	1	0.65
2	2	
3	3	
≥ 4	4	
Vertebrae collapse		
No collapse	1	−0.30
Less than 50%	2	
More than 50%	3	

Notes: The PUFHS was created as follows:

P(Y = 1) = *e*^−2.67 + 0.26*x*1 + 0.65*x*2 − 0.30x3^ / (1+ *e*^−2.67 + 0.26*x*1 + 0.65*x*2 − 0.30x3^); *x* 1 indicates primary cancer types; *x* 2 indicates number of treated vertebrae levels; *x* 3 indicates vertebrae collapse

An example was given as follows: if a spine metastasis patient with rapid growth cancer (3 points) had more than 50% vertebrae collapse (3 points) and one treated vertebra level (1 point), the vascular cement leakage probability of the patient was P (Y = 1) = *e*^−2.67 + 0.26 ∗ 3 + 0.65 ∗ 1 − 0.30 ∗ 3^ / (1+ *e*^−2.67 + 0.26 ∗ 3 + 0.65 ∗ 1 − 0.30 ∗ 3^) = 10.53%

*Abbreviations***:** *PUFHS* Peking University First Hospital Score

^a^indicates characteristics were included according to the Least Absolute Shrinkage and Selection Operator; ^b^indicates estimates were calculated from logistic regression analysis

### Validation of the PUFHS

**Fig. 2:**
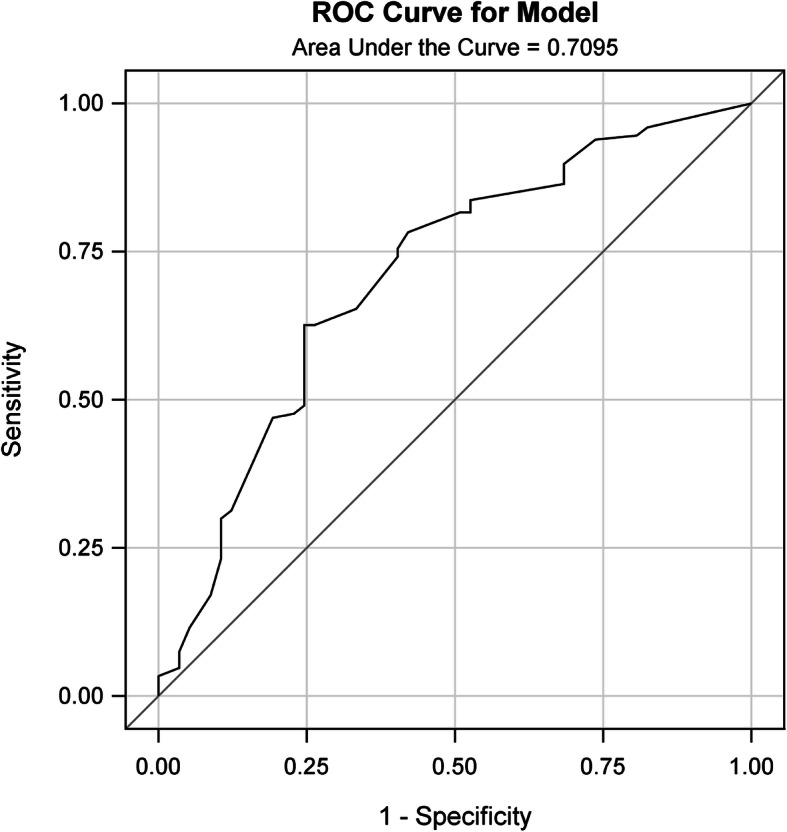
The ROC curve for the PUFHS in the training group (C value = 0.71)

**Fig. 3:**
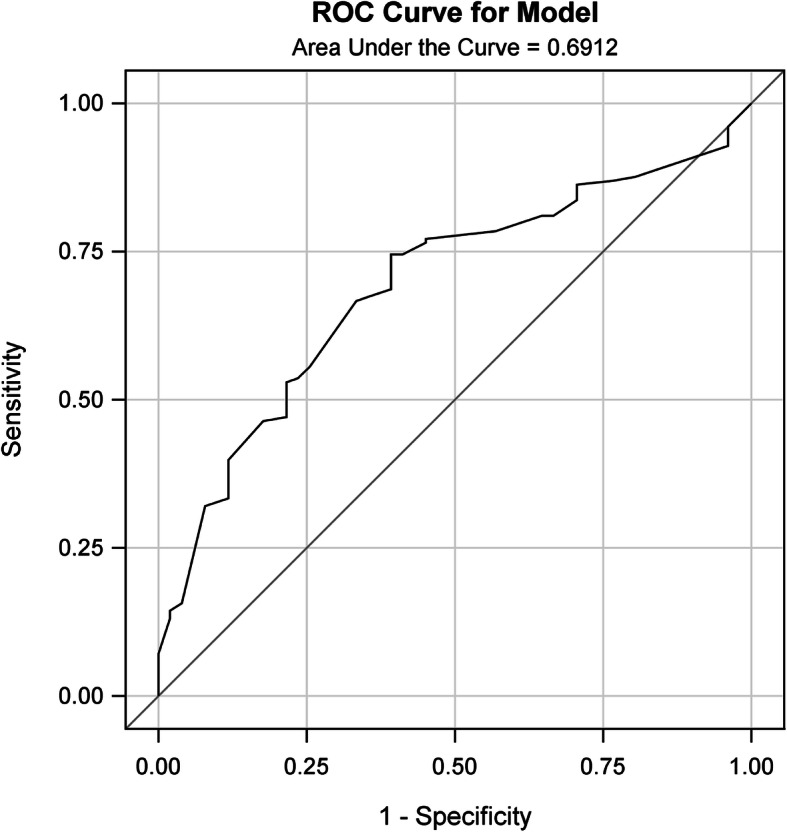
The ROC curve for the PUFHS in the validation group (C value = 0.69)

**Fig. 4:**
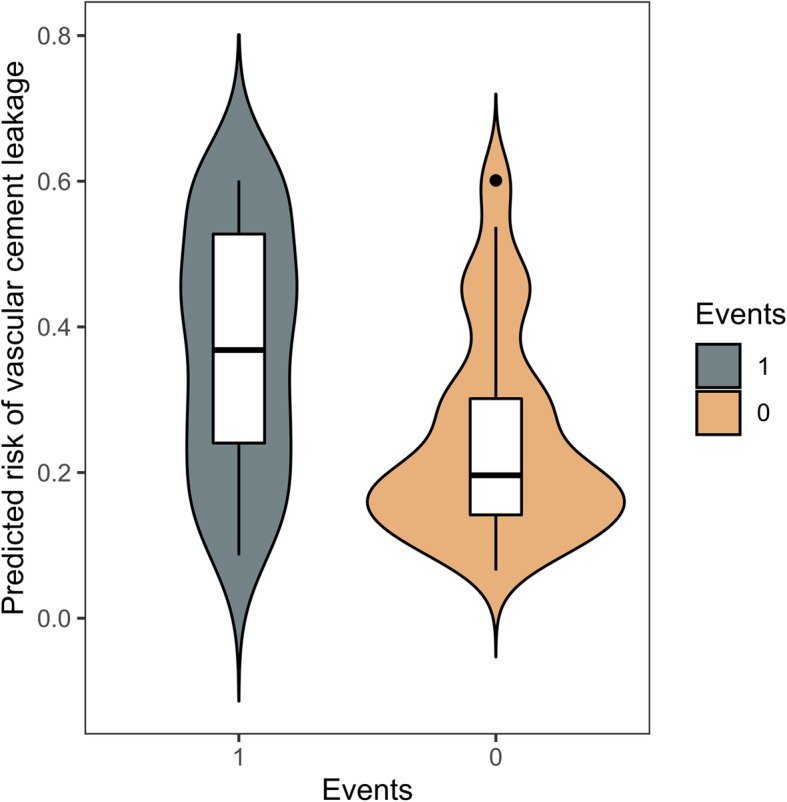
The violin plot for the PUFHS in the training group (Discrimination slope = 0.12, *P* < 0.001). ‘1’ indicates positive event (vascular cement leakage) and ‘0’ indicates negative event (nonvascular cement leakage)

**Fig. 5:**
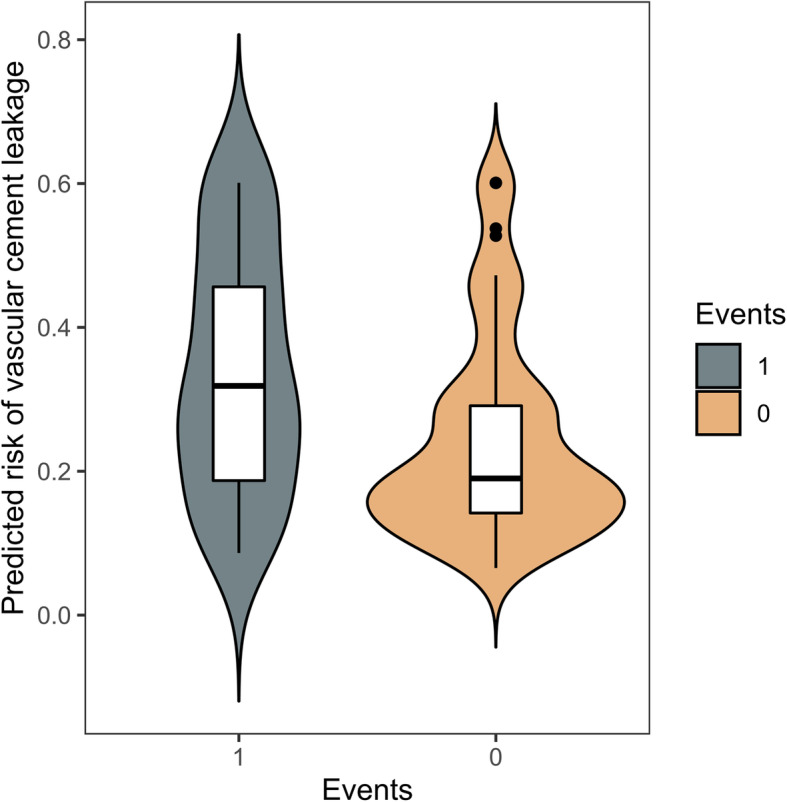
The violin plot for the PUFHS in the validation group (Discrimination slope = 0.096, *P* < 0.001). ‘1’ indicates positive event (vascular cement leakage) and ‘0’ indicates negative event (nonvascular cement leakage)

Discrimination and calibration were performed both in the training and validation group. The AUROC was 0.71 in the training group (Fig. 2) and 0.69 in the validation group (Table 4 and Fig. 3), which demonstrated the PUFHS was a useful and good model. The corresponding correct classification rates were 73.0 and 70.1%, respectively. The discrimination slope was 0.12 (95% CI: 0.078–0.17, *P* < 0.001) in the training group (Fig. 4) and 0.096 (95% CI: 0.049–0.14, *P* < 0.001) in the validation group (Fig. 5), which indicated the mean risk differences between positive and negative events were significant in the two groups. The sensitivity and specificity was 24.6 and 91.8%, respectively, in the training group and 29.4 and 83.7%, respectively, in the validation group.

**Table 4:** The discrimination performances of the PUFHS in the training and validation group

Evaluation analysis	AUROC	CCR	Slope	95% CI	Sensitivity	Specificity
The training group	0.71	73.0%	0.12	0.078–0.17	24.6%	91.8%
The validation group	0.69	70.1%	0.096	0.049–0.14	29.4%	83.7%

*Abbreviations***:** *PUFHS* Peking University First Hospital Score, *AUROC* area under the receiver operating characteristic curve, *CCR* correct classification rate, *CI* confidence interval

**Fig. 6:**
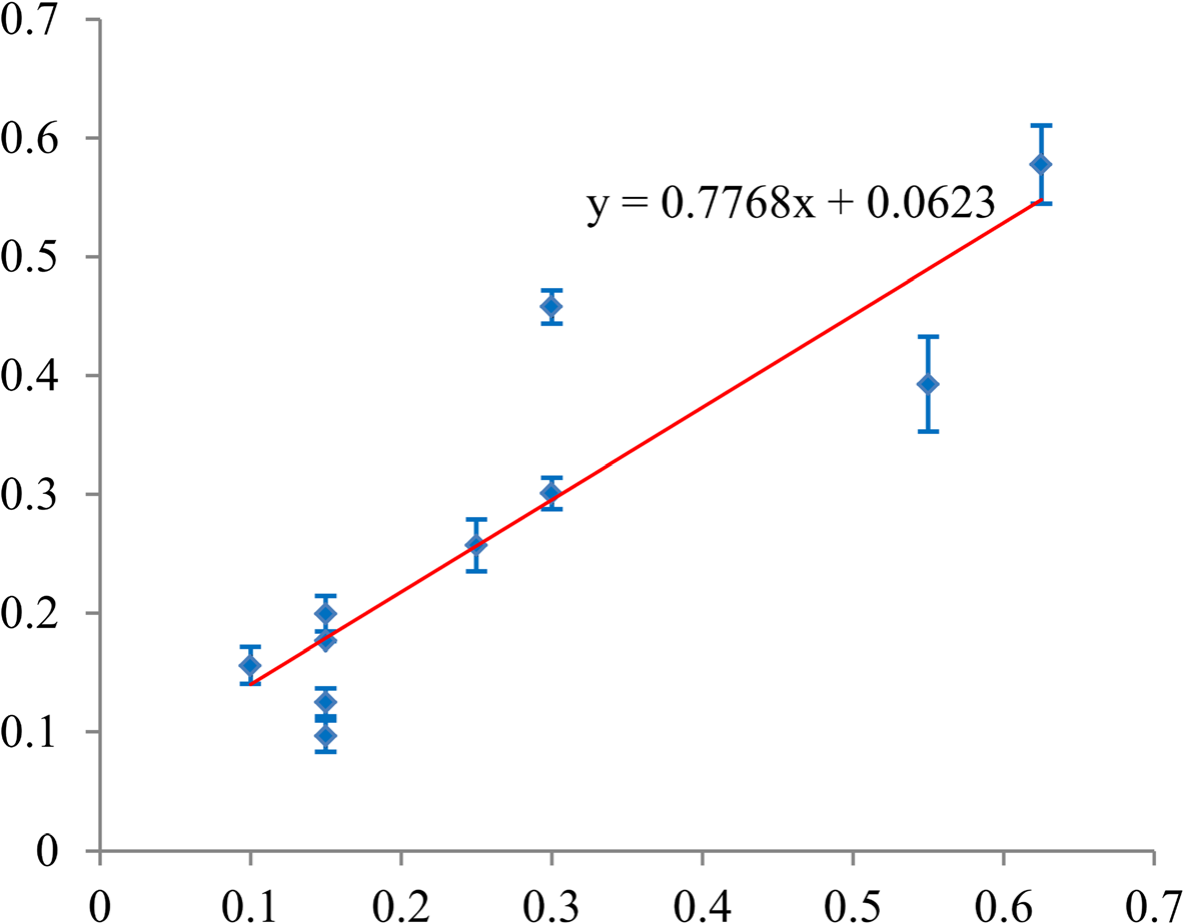
Plotting decile of the PUFHS-predicted probability of vascular canal cement leakage against the observed proportions in the training group (Calibration slope = 0.78 and intercept = 0.06). The red line indicates the smooth was fitted using linear regression analysis in the Microsoft Excel software

**Fig. 7:**
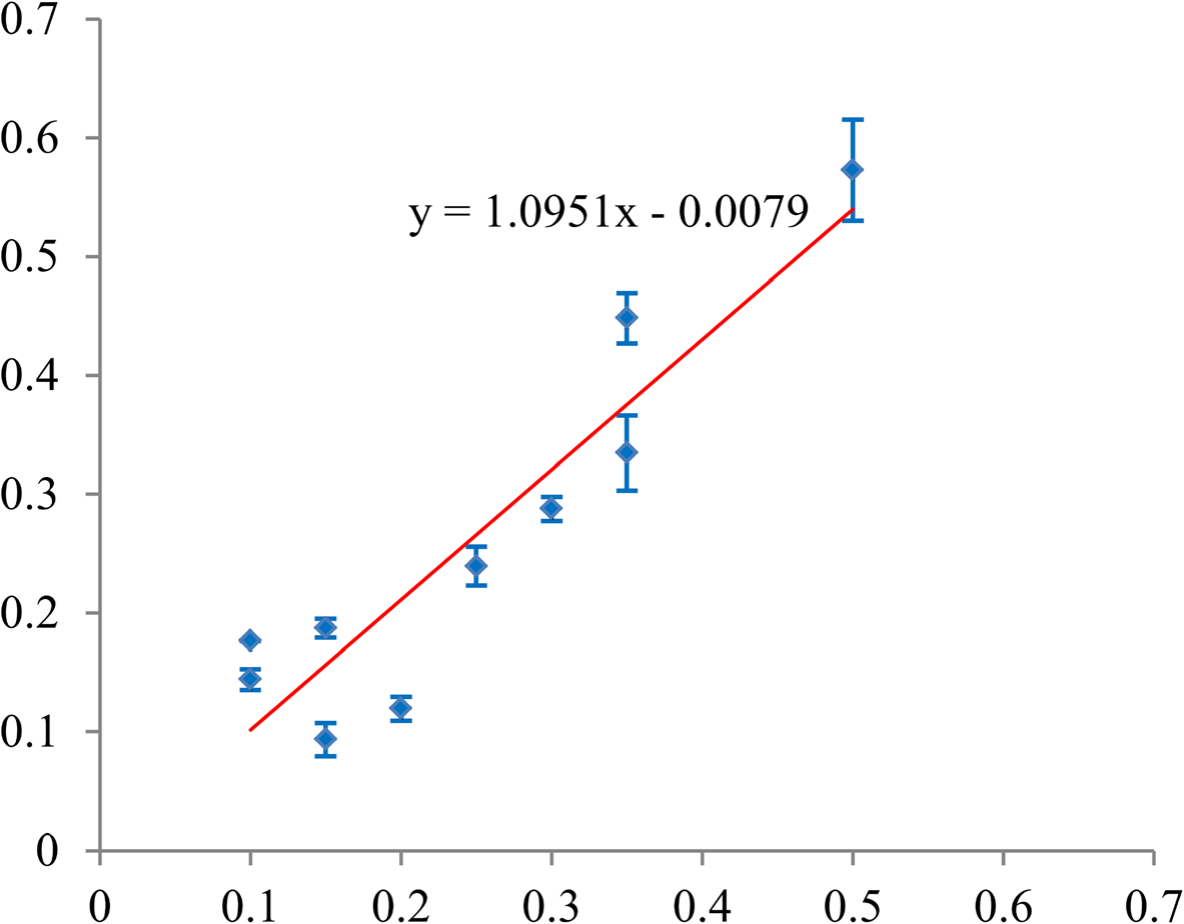
Plotting decile of the PUFHS-predicted probability of vascular canal cement leakage against the observed proportions in the validation group (Calibration slope = 1.10 and intercept = − 0.0079). The red line indicates the smooth was fitted using linear regression analysis in the Microsoft Excel software

Considering the calibration ability, the slope was 0.78 (95% CI: 0.45–1.10) in the training group (Fig. 6) and 1.10 (95% CI: 0.73–1.46) in the validation group (Table 5 and Fig. 7). The corresponding intercepts were 0.06 (95% CI: − 0.04–0.17) and − 0.0079 (95% CI: − 0.11–0.092), respectively, both of which were close to 0. The *P*-values obtained from the goodness-of-fit test were both more than 0.05 in the two groups. These results, as mentioned above, indicated the PUFHS had good calibration ability. Table 6 shows the observed and predicted probability according to decile in the training and validation group. According to the predicted probabilities in each decile, patients were divided into three risk groups: the low, medium, and high group. The low risk group had a probability of less than 20% for vascular cement leakage, the medium risk group had a probability of 20% or more and less than 40%, and the high risk group had a probability of 40% or more. The observed and predicted probabilities of vascular cement leakage were both significant different among the three risk groups (*P* < 0.01).

**Table 5:** The calibration performances of the PUFHS in the training and validation group

Evaluation analysis	Slope	95% CI	Intercept	95% CI	Goodness-of-Fit test
The training group	0.78	0.45–1.10	0.06	−0.04–0.17	0.98
The validation group	1.10	0.73–1.46	−0.0079	−0.11–0.092	0.16

*Abbreviations***:** *PUFHS* Peking University First Hospital Score, *CI* confidence interval

**Table 6:** Observed and predicted probability according to decile in the training and validation group

Groups	Probability	Decile									
		1th	2nd	3rd	4th	5th	6th	7th	8th	9th	10th
The training group	Observed	15.00%	15.00%	10.00%	15.00%	15.00%	25.00%	30.00%	55.00%	30.00%	62.50%
	Predicted (Mean ± SD)	9.64% ±1.33%	12.50% ±1.20%	15.60% ±1.56%	17.65% ±0.00%	19.94% ±1.51%	25.69% ±2.19%	30.10% ±1.33%	39.27% ±3.98%	45.79% ±1.40%	57.78% ±3.31%
The validation group	Observed	15.00%	20.00%	10.00%	10.00%	15.00%	25.00%	30.00%	35.00%	35.00%	50.00%
	Predicted (Mean ± SD)	9.36% ±1.40%	11.96% ±1.02%	14.41% ±0.88%	17.65% ±0.00%	18.72% ±0.81%	23.94% ±1.64%	28.77% ±1.01%	33.46% ±3.16%	44.83% ±2.12%	57.28% ±4.26%
Patients (n)		20	20	20	20	20	20	20	20	20	24
Risk groups		The low risk group (≧0 and < 20%)					The medium risk group (≧20 and < 40%)			The high risk group (≧40%)	
Observed probability (the validation group)		14.47% ± 3.63%					29.24% ± 4.67%			52.18% ± 6.78%	
P^*^		< 0.01									
Predicted probability (the validation group)		13.86% (14/101)					29.51% (18/61)			45.24% (19/42)	
*P*^†^		< 0.01									

^*^indicates the Kruskal Wallis test; ^†^indicates the Chi-square test

## Discussion

The study found three risk predictors, namely, primary cancer types, number of treated vertebrae levels, and vertebrae collapse, were significantly associated with vascular cement leakage in spine metastases following percutaneous vertebroplasty. We further created the PUFHS with based on the above three risk predictors. The PUFHS was simple since it had only three variables. The predictive performance of the PUFHS was evaluated both in the training and validation group. The AUROC was 0.71 in the training group and 0.69 in the validation group, which demonstrated the PUFHS was a good and useful model. The calibration slope was near to 1 (0.78 in the training group and 1.10 in the validation group) and the intercepts (0.06 in the training group and − 0.0079 in the validation group) were close to 0, which revealed the PUFHS also had good calibration ability. Therefore, the PUFHS can be a useful tool to realize early detection of vascular cement leakage and inform surgeons about the risk in advance. Furthermore, we developed a calculator which can calculate the probability of vascular cement leakage in order to facilitate the clinical utility of the score (see Supplementary material). Based on the predicted probabilities, patients were divided into three risk groups: the low, medium, and high groups. Among the three groups, the high risk group had the highest probability of vascular cement leakage (40% or more), thus careful surgical preparation and intraoperative operation should be especially emphasized in those patients.

Of all the patients in the study, vascular cement leakage occurred in 26.47% of patients and this number was consistent with other studies. Corcos et al. [15] reported 25% of patients had vascular cement leakage after analyzing 56 cancer patients. Trumm et al. [16] found 25.5% of treated vertebrae occurred vascular leaks into segmental veins and 21.6% leaks into basivertebral veins after analyzing 202 malignant tumor patients. Pulmonary cement embolisms were observed after 7.8% of the procedures with follow-up of the X-ray of chest. Notably, vascular cement leakage was strongly associated with pulmonary embolism [17]. However, Barragán-Campos et al. [17] reported 423 cement leakages were identified in 117 patients and 78.5% of them were vascular. Inherent heterogeneity among spine metastatic lesions and technical diversity including PMMA-injecting volume, flow, and viscosity could lead to the difference.

Some studies also reported several risk factors were significantly associated with vascular cement leakage. Corcos et al. revealed [15] prior treatment and vertebral collapse were correlated with vascular cement leakage. Our study also found vertebral collapse was inversely correlated with vascular cement leakage, which was consistent with the study conducted by Corcos and his colleagues [15]. However, preoperative treatment was not found to be significant in our study. Corcos and his colleagues reckoned reduction in intravertebral pressure and vertebral vascularity after previous treatments could explain the role of prior treatment in preventing vascular cement leakage. We speculated heterogeneous definition of prior treatment might cause the difference.

In our study, primary cancer type was included in the PUFHS. Rapid growth cancers were more likely to suffer from vascular cement leakage. Reidy et al. [18] found vertebrae containing simulated metastatic tumor could significantly increase intravertebral body pressures during percutaneous vertebroplasty as compared with intact vertebrae. Higher intravertebral body pressures could lead to more cement leakages. Besides, vertebral vascularity could definitely affect vascular cement leakage since hypervascularity provided more ways for PMMA to leak. Thus, high intravertebral pressures, resulted from rapid growth of metastatic cancers, and abundant vertebral vascularity could justify the results. The number of treated vertebrae segments was also proved to be significant simply because the more surgically treated segments indicated the greater the possibility of cement leakage.

Previously, we proposed an algorithm based on the treated vertebrae level, cortical osteolytic destruction in the posterior wall, and the Bilsky scale, which can calculate cement injection volumes in spine metastases treated with percutaneous vertebroplasty [19]. This algorithm can help surgeons to guide surgical planning and cement injections. However, the algorithm still cannot early predict cement leakage and thus prevention strategies cannot be performed in advance. In the present study, we created the PUFHS, making early detection of vascular cement leakage a reality. Besides, classifying patients in the low, medium, and high risk group contributes to enhanced quality of healthcare. We aimed at developing an algorithm especially to calculate the probability of vascular cement leakage because the multitude of patients only had one vertebra metastasis and sometimes we found vascular cement leakage in the vein but we cannot distinguish which vertebra the cement leakage came from particularly in cases treating with multiple vertebra percutaneous vertebroplasty. Thus we performed the analysis per patient rather than per vertebra, this might add precision.

This study had several limitations. First, the study was retrospective and enrolled patients in a single medical center, so selection bias would definitely exist. Second, some risk variables, such as PMMA-injecting volume, flow, and viscosity, which could influence vascular cement leakage [20], were not assessed in the study. Therefore, although the PUFHS showed good discrimination and calibration ability, the PUFHS still need large prospective sample to be validated.

## Conclusion

Vascular cement leakage is common among spine metastases after percutaneous vertebroplasty. The PUFHS can calculate the probability of vascular cement leakage, which can be a useful tool to inform surgeons about vascular cement leakage risk in advance.

## Supplementary Information

**Additional file 1.**

## Data Availability

The datasets of the current study are available under reasonable request.

## Abbreviations

AUROC: Area under the receiver operating characteristic curve
CCR: Correct classification rate
CI: Confidence interval
PUFHS: Peking University First Hospital Score
LASSO: Least Absolute Shrinkage and Selection Operator
N.A: Not applicable
SD: Standard deviation
